# DrGA: cancer driver gene analysis in a simpler manner

**DOI:** 10.1186/s12859-022-04606-0

**Published:** 2022-03-05

**Authors:** Quang-Huy Nguyen, Tin Nguyen, Duc-Hau Le

**Affiliations:** 1 School of Computer Science and Engineering, Thuyloi University, Hanoi, Vietnam; 2 Department of Computer Science and Engineering, University of Nevada, Reno, NV USA

**Keywords:** Driver gene, Genetic biomaker, Human breast cancer, Mouse metabolic syndrome, Clinical feature, Omics data

## Abstract

**Background:**

To date, cancer still is one of the leading causes of death worldwide, in which the cumulative of genes carrying mutations was said to be held accountable for the establishment and development of this disease mainly. From that, identification and analysis of driver genes were vital. Our previous study indicated disagreement on a unifying pipeline for these tasks and then introduced a complete one. However, this pipeline gradually manifested its weaknesses as being unfamiliar to non-technical users, time-consuming, and inconvenient.

**Results:**

This study presented an R package named DrGA, developed based on our previous pipeline, to tackle the mentioned problems above. It wholly automated four widely used downstream analyses for predicted driver genes and offered additional improvements. We described the usage of the DrGA on driver genes of human breast cancer. Besides, we also gave the users another potential application of DrGA in analyzing genomic biomarkers of a complex disease in another organism.

**Conclusions:**

DrGA facilitated the users with limited IT backgrounds and rapidly created consistent and reproducible results. DrGA and its applications, along with example data, were freely provided at https://github.com/huynguyen250896/DrGA.

**Supplementary Information:**

The online version contains supplementary material available at 10.1186/s12859-022-04606-0.

## Background

To date, cancer ranks 2nd among the world's causes of death behind cardiovascular disease [1]. Genes carrying mutations are a potential culprit for establishing and developing cancer [2–6]. This is a strong motivation pushing cancer researchers to identify and analyze cancer-associated genes [7–15], possibly advancing cancer therapeutics. Our previous work [16] have shown disagreement on a unifying pipeline for cancer driver identification and analysis, and then introduced a complete one with two main contributions: (1) collection of the most widely used analysis steps with advanced statistical tools in the field, and (2) reasonable selection of the best parameters of those tools for each particular case.

However, our pipeline gradually manifests its weaknesses. It may take time for beginners interested in this field but unfamiliar with programming (i.e., they must learn complex concepts and run lengthy R codes). Besides, we realize that all the methods are web-based apps or R packages that may lead the users to a little inconvenience while using our pipeline. Moreover, although many driver gene identification tools have been proposed, driver gene analysis tools have been minimal, generally integrated with the identification tools and only focusing mainly on enrichment analysis [17–19]. Inspired by these, we have built DrGA based on the aforementioned pipeline as a solution to simplify the analysis process. In particular, DrGA offers several additional improvements, including an automatic implementation for analyses in R only and the best settings/parameters are automatically but flexibly selected case-by-case. These help cancer researchers at different programming skill levels to effortlessly issue consistent and reproducible results.

In this study, we present two applications of the DrGA on two case studies of human breast cancer and mouse metabolic syndrome using multi-omics datasets. We hypothesize that DrGA with high-end tools that support the individual- and system-level analyses will be efficient in characterizing cancer driver genes as well as genetic biomarkers.

### Implementation

**Fig. 1:**
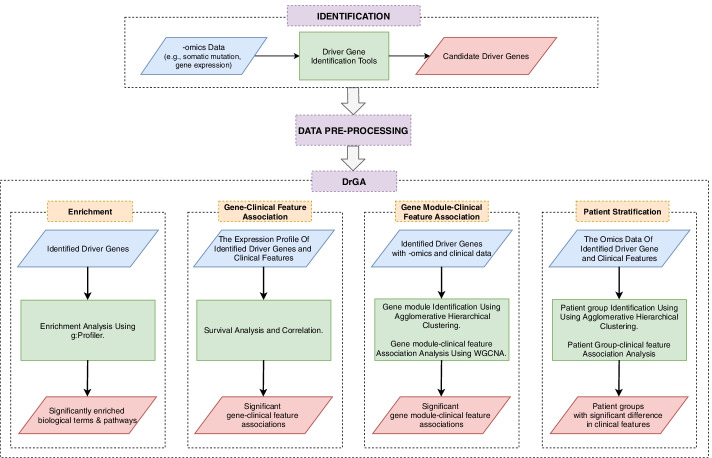
Framework of DrGA. DrGA, armed with the four widely used analyses, dealt automatically with identified driver genes, and then provided the users with analysis results moved directly to predefined R working directory or printed out in R console results

Figure 1 illustrates a four-module framework of DrGA, including enrichment analysis, individual gene-clinical feature association analysis, functional module-clinical feature association analysis, and patient stratification, to discover driver genes. At first, DrGA functionally enriched the candidate drivers using R package gprofiler2 [20] (module 1). Then, it further investigated the associations between expression levels of each gene versus each clinical feature of choice (e.g., tumor stages, weight, glycemic index,…), and versus patient outcomes as well (module 2). In parallel, DrGA also performed the association analyses of functional gene modules (identified by an agglomerative hierarchical clustering [21]) with those clinical features using an improved version of WGCNA [22] proposed by us before [16] (module 3). At last, the tool clustered samples using all the identified driver genes from -omics data (e.g., copy number alteration, or methylation, or gene expression, or the like) using the same clustering method (module 4). Given the user’s -omics data, they now only detected driver genes using advanced driver gene identification tools and then processed data following an easy-to-meet format required by DrGA (see Additional file 1). All detailed comparisons of methods/tools included in each DrGA’s module with other state-of-the-art techniques as well as previously proposed improvements were thoroughly discussed in the original paper [16]. Here, aside from summarizing important changes proposed in [16], we also indicated that DrGA offered several additional improvements.

### Module 1: enrichment analysis

In our prior study [16], we recommended the users to choose g:Profiler [20] instead of GSEA [23], DAVID [24, 25], Gene Ontology [26], KEGG [27] or IPA [28] for this task since it was the rich-annotated, friendly web-based, freely used, and monthly up-to-date enrichment analysis source. However, we then realized that it would be inconvenient for the users to use multiple platforms (web-based apps and R-packages) when dealing with their set of predictive driver genes. From that, we decided to integrate an R-package, gprofiler2 [20], into DrGA, and so DrGA was able to automatically analyze enrichment on those driver genes. Especially, to apply DrGA to any organism, e.g., human, mouse, yeast, etc., the users only needed to use the argument ‘organism’. The analysis results of module 1 would move to the predefined working directory as a txt file. DrGA considered the driver gene as significantly enriched with GO terms and pathways if Q-value ≤ 0.05 (g:SCS multiple testing correction method [20], two-sided).

### Module 2: association analyses

We previously indicated that selecting the P-value adjustment method was not often specified, resulting in having difficulty reproducing analysis results, such as ref. [11]. Therefore, DrGA now included the Benjamini–Hochberg procedure [29] and automatically analyzed associations between the expression levels of each driver gene versus each clinical feature of interest and versus patient survival, rendering respective analysis results formatted as txt files and placed at the user’s predefined R working directory.

The users could use different correlation methods by feeding one of the three optional choices: pearson, spearman, or kendall to the argument ‘methodCC’ (i.e., Pearson’s correlation, Spearman’s rank-order correlation or Kendall’s tau correlation, respectively) to perform associations between expression levels of each driver gene and each clinical feature of interest over samples. The Pearson’s /Spearman’s /Kendall’s coefficients (*r*) of each driver gene with one clinical feature of choice were calculated as follows (Eq. 1–3):

1 $$r_{p} = \frac{{\mathop \sum \nolimits_{i = 1}^{n} \left( {X_{i} - \overline{X}} \right)\left( {Y_{i} - Y} \right)}}{{\sqrt {\mathop \sum \nolimits_{i = 1}^{n} \left( {X_{i} - \overline{X}} \right)} \sqrt {\mathop \sum \nolimits_{i = 1}^{n} \left( {Y_{i} - \overline{Y}} \right)} }}$$

2 $$r_{s} = \frac{{\mathop \sum \nolimits_{i = 1}^{n} \left( {rx_{i} - \overline{rx} } \right)\left( {ry_{i} - \overline{ry} } \right)}}{{\sqrt {\mathop \sum \nolimits_{i = 1}^{n} \left( {rx_{i} - \overline{rx} } \right)} \sqrt {\mathop \sum \nolimits_{i = 1}^{n} \left( {ry_{i} - \overline{ry} } \right)} }}$$

3 $$r_{k} = \frac{C - D}{{C + D}}$$

where *r*_*p*_, *r*_*s*_, and *r*_*k*_ were the Pearson, Spearman, and Kendall’s correlation coefficients, respectively; n was number of pairs; X_i_ and Y_i_ were the ith expression level of a driver gene and ith value of a clinical feature of choice over patients; $\overline{x }$ and $\overline{y }$ were the mean expression level of that driver gene and mean value of that clinical feature over patients. rx_i_ and ry_i_ were the ith ranked expression levels of a driver gene and ith ranked values of a clinical feature of choice over patients, $\overline{rx }$ and $r\overline{y }$ were the average rank of expression levels of that driver gene and average rank of values of that clinical feature over patients. C and D were the number of concordant pairs and discordant pairs, respectively.

DrGA also helped the users analyze the prognostic effects of predicted driver genes automatically. The tool required gene expression profiles, divided into two groups: up or down expression groups based on mean/median expression levels, and survival information of patients included in the clinical data as input. Then, a log-rank test in univariate Cox regression analysis with a proportional hazards model [30] was performed to compute those associations. Next, hazard ratios (HR) with their 95% confidence intervals (CI), Cox P-values, and Q-values were recorded and reported as a txt file. DrGA considered the driver gene as significantly associated with survival rate if Q-value ≤ 0.05 (Benjamini–Hochberg procedure, two-sided).

### Module 3: construction of co-expressed gene modules

**Fig. 2:**
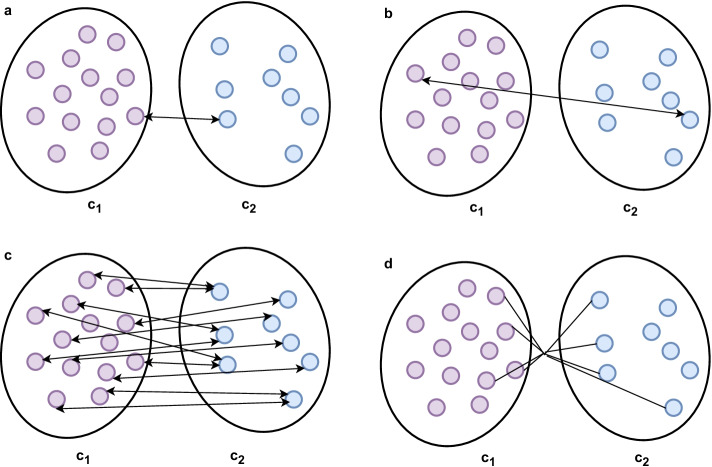
Illustration of four agglomeration methods included in DrGA. The number of subgroups was two for example purpose. **a** single-linkage method. **b** complete linkage method. **c** average-linkage method. **d** Ward’s minimum variance method. *c*_*1*_* and c*_*2*_, cluster 1 and cluster 2

Numerous existing studies have reported that multifactorial diseases, like cancer, have been caused by a group of genes instead of individual genes [31, 32]. Besides, gene co-expression networks are one of the most common ways to reveal a collection of genes functioning collaboratively [33, 34] as well as a collection of hub genes that are of utmost importance in a certain disease, and that WGCNA is a pioneer in this problem. Basically, WGCNA attempts to build co-expressed modules of genes based on a gene–gene similarity matrix across a group of patients having a tendency to show a coordinated expression pattern [22]. Our previous study [16] introduced an improved version of WGCNA, temporarily called iWGCNA in this study, and confirmedly outperformed its original version in the ability to detect functional gene modules [35]. Specifically, we predetermined which cluster distance measure, including the single-linkage, complete-linkage, average-linkage, or Ward’s minimum variance [36] methods (Table 1 and Fig. 2), was appropriate for each particular case based on agglomerative coefficients, helping measure the number of clustering structures found and specify the agglomeration method to be used. To simplify this process, DrGA was now able to do this task automatically. Next, Pearson’s correlation coefficients and corresponding P-values between each identified co-expressed module and clinical features of choice were computed automatically and outputted as a publication-quality figure in PDF format. Also, DrGA automatically reported the top-five hub genes (i.e., genes with high intramodular connectivity) in each co-expressed module, indicating possession of a vast range of interactions with other genes as well as playing a crucial role in the co-expression network of those genes.

**Table 1:** Four agglomeration methods considered automatically in DrGA to specify the appropriate one

Cluster distance measure	Description	Formula
Single method	The distance between two clusters, c_1_ and c_2_, is defined as the shortest distance between two points, x_1_ and x_2_ in each cluster	$D\left( {c_{1} ,c_{2} } \right){ } = { }\mathop {\min }\limits_{{x_{1} \in c_{1} , x_{2} \in c_{2} }} D\left( {x_{1} ,x_{2} } \right)\quad \quad (4)$
Complete method	The distance between two clusters, c_1_ and c_2_, is defined as the longest distance between two points, x_1_ and x_2_ in each cluster	$D\left( {c_{1} ,c_{2} } \right){ } = { }\mathop {\max }\limits_{{x_{1} \in c_{1} , x_{2} \in c_{2} }} D\left( {x_{1} ,x_{2} } \right)\quad \quad (5)$
Average method	The distance between two clusters, c_1_ and c_2_, is defined as the average distance between each point in one cluster to every point in the other cluster	$D\left( {c_{1} ,c_{2} } \right){ } = { }\frac{1}{{n_{c1} n_{c2} }}\mathop \sum \limits_{i = 1}^{{n_{c1} }} \mathop \sum \limits_{j = 1}^{{n_{c2} }} D\left( {x_{i} ,x_{j} } \right)\quad \quad (6)$
Ward’s method	Minimizes the total within-cluster error sum of squares, and then, at each stage, iteratively identifies pairs of groups with minimum between-group distance and carry out the merger of those two	$TD_{{c_{1} \cup c_{2} }} = \mathop \sum \limits_{{x \in c_{1} \cup c_{2} }} D\left( {x,\mu_{{c_{1} \cup c_{2} }} } \right)^{2} \quad \quad (7)$

*D(X,Y)* the distance between X and Y, *c*_*1*_* and c*_*2*_ cluster 1 and cluster 2, *x*_*1*_* and x*_*2*_ a point in cluster 1 and a point in cluster 2, *TD*total distance, $\mu$ mean

### Module 4: hierarchical clustering of cancer patients

Many works [11, 14] used the agglomerative hierarchical clustering technique at a basic level to partition the cancer patients into different subgroups that could be improved. Indeed, similar to module 3, DrGA also re-determined automatically which agglomeration method was optimal. Besides, one important task in the clustering problem was how many subgroups were optimal. Prior works sometimes ignored this step or made it ambiguous [11]. To solve this problem, DrGA automatically and simultaneously implemented three common indices: the average Silhouette index [37], the Dunn’s index [38, 39], and the connectivity. The average Silhouette took a value between − 1 (poorly clustered observations) and 1 (well clustered observations), and the place where the black line of the Silhouette plot peaks at, which implied that that subgroup number was optimal. The Dunn’s index took a value between zero (poorly clustered observations) and infinity (well clustered observations), and the place where the black line of Dunn’s index plot peaks at, which implied that that group number was optimal. The connectivity showed the connectedness of a given cluster partitioning and took a value between 0 and infinity. The user should choose a point reaching the most minimized value. Figure 4 shows gained results for illustrative purpose. In practice, the optimal number of subgroups would be the number of being selected by the three indices. If not, two out of the three indices; otherwise, DrGA would report that it did not find any optimal number (a sporadic case).

To examine possible differences between involved subgroups in patient survival and clinical features, DrGA first automatically implemented survival analysis using the Cox regression between the identified subgroups, and outputted the P-value and the HR with its 95% CI in the R environment. Then, DrGA also automatically performed comparison between the identified subgroups in terms of the selected clinical features using statistical tests. The results were moved into the working directory as an xlsx file. Noticeably, DrGA automatically recognized whether those clinical features were continuous normal-distributed, continuous non-normal distributed, or categorical to select an appropriate statistical test. The way to let DrGA be able to do that was by using the Shapiro–Wilk test for normality [40]. Given a certain clinical feature, the null hypothesis H_o_ was that the clinical feature followed a normal distribution. Then if the *P* value ≤ 0.05, DrGA rejected H_o_.

Note that we discussed exhaustedly how to use DrGA and interpret the results in the section ‘Understanding the tool and gained results’ in Additional file 1.

## Results

### Human breast cancer

Here we re-used-omics data used in our prior study [16], downloaded from the cBioPortal for Cancer Genomics (http://www.cbioportal.org) [41, 42], including somatic mutation, gene expression, and copy number alterations, in a cohort of breast cancer patients. We decided to apply DrGA to these datasets to demonstrate that DrGA were well able to reproduce all the results indicated in [16] but in a surprisingly rapid way. More details of the pre-processing procedures and analysis processes could be found in the Additional file 1. As expected, we inputted processed data into DrGA and received the same results relative to [16] in only about 30 s.

### Mouse metabolic syndrome

Here we strived to go beyond the initial goal of DrGA with an example of mouse metabolic syndrome (obesity, insulin resistance, and dyslipidemia) [43]. The data were gene expressions in the liver from female mice and a set of physiologically relevant genes. In this section, to avoid using too many clinical features for the analysis process, we also added a corresponding mini-step in pre-processing procedures (Additional file 1: Fig. S15). As a result, eight out of 20 physiological features were kept, including bodyweight, body length, abdominal fat, total fat, ulcerative colitis, free fatty acids, glycemic index, and two LDL and VLDL cholesterol levels.

**Fig. 3:**
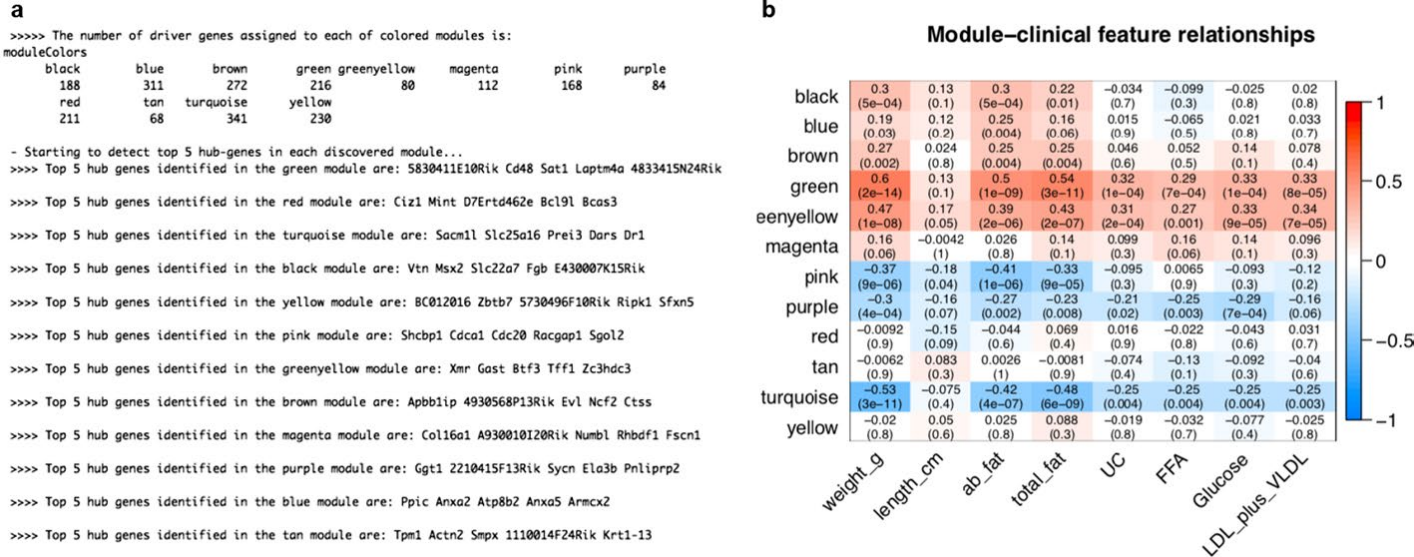
Analysis results performed in module 3 of DrGA. **a** DrGA discovered 12 co-expressed modules with corresponding numbers of genes as well as top-five hub genes included in each module. **b** Associations between each module and the eight selected clinical features. *weight_g*, bodyweight of mice (gram unit), *length_cm* body length of mice (centimeters unit), *ab_fat* abdominal fat, *total_fat* total fat, *UC* ulcerative colitis, *FFA* free fatty acids, *Glucose* glycemic index, *LDL_Plus_VLDL* two LDL and VLDL cholesterol levels

Full findings of DrGA, in this case, could be found in our Github (https://github.com/huynguyen250896/DrGA). Here we wanted to discuss more the most interesting results than the analysis results of [43]. As shown in Fig. 3a, DrGA discovered 12 co-expressed gene modules, consistent with the module number reported in [43], and the top-five hub genes were detected automatically and printed out in the R console results. These genes were extremely interesting since it was evident that genes with very high connectivity in lower organisms were confirmedly associated with lethal phenotypes [44–46]. In addition, Fig. 3b reports genes belonging to the green module were jointly expressed, which resulted in the most positive correlation with the syndrome. The opposite was seen in the turquoise module.

**Fig. 4:**
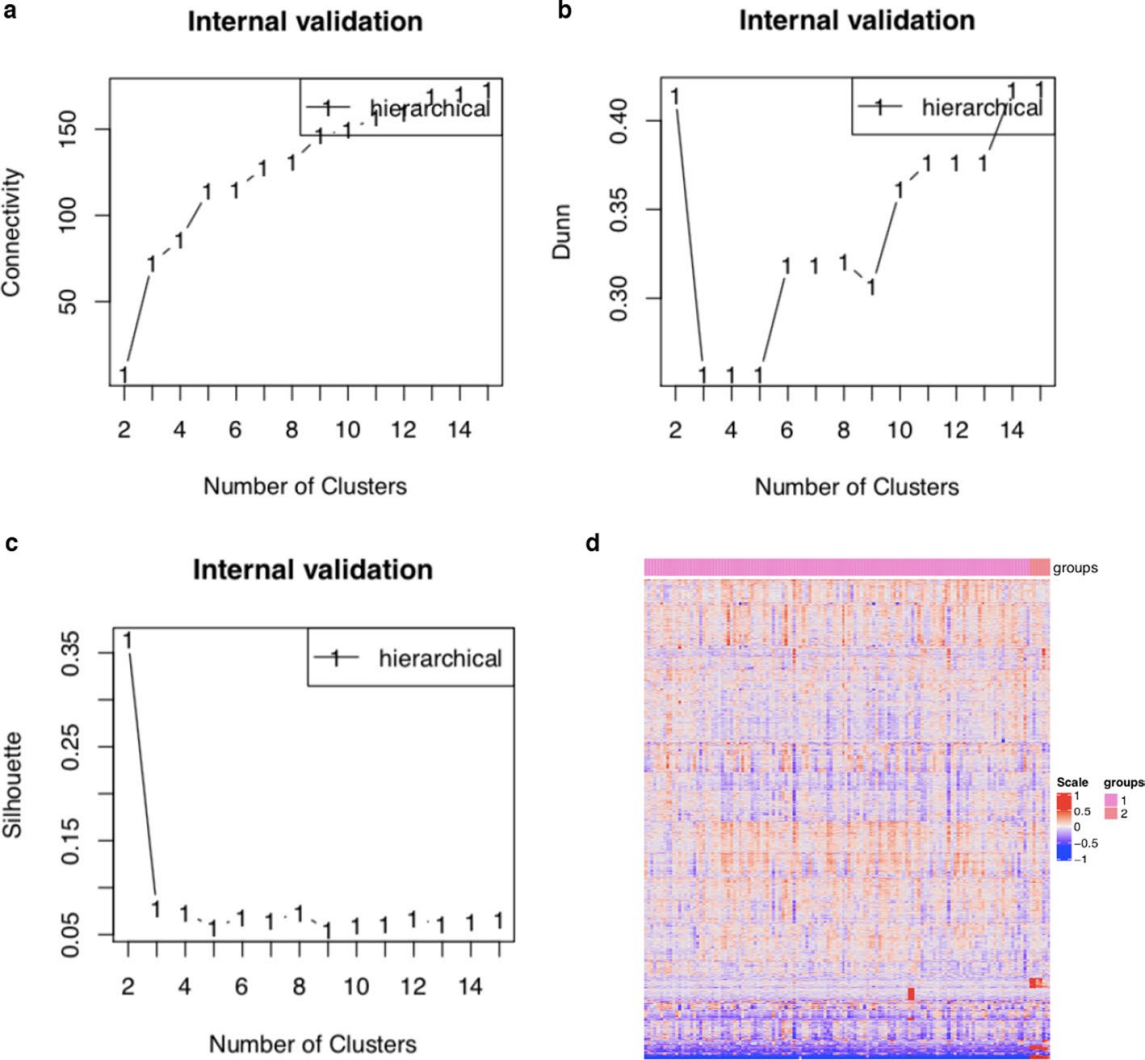
Identification of the optimal number of subgroups. **a** Connectivity index selected two subgroups. **b** Dunn index selected two subgroups. **c** Silhouette index selected two subgroups. **d** Differences in expression events between the identified groups. Two distinct groups were found (pink and orange)

Next, DrGA tried stratifying the mice using the methodology described in the Methods section. As a result, all the three indices reported the two subgroups were optimal (Fig. 4a-c). Figure 4d shows the heatmap illustrating differences in expression events between the included subgroups. Finally, the comparisons between these subgroups in terms of the eight clinical features of choice were implemented automatically by DrGA (Table 2). Unfortunately, we did not see any statistically significant differences in the selected clinical features between the two subgroups, most possibly due to the small number of samples. However, we still saw that mice assigned to the first subgroup had partially significantly worse traits than their counterparts in the second subgroup (higher weight, higher total fat, higher free fatty acids levels, and higher glycemic index).

**Table 2:** Comparison between the involved subgroups in terms of the chosen clinical features

	1 (N = 125)	2 (N = 7)	*p* value
weight_g	38.2 (6.21)	36.5 (2.24)	0.110
length_cm	10.2 (0.34)	10.2 (0.36)	1.000
ab_fat	2.53 [1.74;3.20]	2.04 [1.86;2.27]	0.268
total_fat	4.91 [3.97;5.86]	3.96 [3.55;4.19]	0.059
UC	460 (122)	417 (122)	0.401
FFA	109 (29.0)	86.0 (28.7)	0.079
Glucose	432 (97.4)	375 (71.9)	0.086
LDL_plus_VLDL	1196 (315)	1103 (246)	0.371

For the first two continuous variables: weight_g and length_cm, and the last four continuous variables: UC, FFA, Glucose, LDL_plus_VLDL, median [percentiles 25%; percentiles 75%] were calculated at the first two columns. For the remaining two ordinal variables: ab_fat and total_fat, the number of cases and the percentage of cases in each tumor stage are shown

weight_g, bodyweight of mice (gram unit); length_cm, body length of mice (centimeters unit); ab_fat, abdominal fat; total_fat, total fat; UC, ulcerative colitis; FFA, free fatty acids; Glucose, glycemic index; and LDL_Plus_VLDL, two LDL and VLDL cholesterol levels

For this moment, it took us only about two minutes to finish all the analysis modules.

## Discussion and future work

We have described DrGA with functions in a more straightforward manner and shown its ability through the two benchmark datasets, including human breast cancer and mouse metabolic syndrome. We also have described our recent modifications to DrGA, which served to improve both its usability as well as its ability to keep analysis results consistent and reproducible without recourse to programming expertise. Besides, we also open up another potential application of DrGA on complex diseases from other species, proving DrGA is very flexible to characterize driver genes or genomic biomarkers and be applied to any organism such as human, mouse, yeast, etc. These will help expand the pool of users with different backgrounds, including biologists, bioinformaticians, and computational biologists, in analyzing cancer genes and biomarkers from –omics data.

Nevertheless, we acknowledge that DrGA has still several limitations. Firstly, DrGA automatically performs the correlation analysis just using the three commonly used methods (i.e., Pearson’s, Spearman’s rank, and Kendall’s tau correlations), which might lead to forcing the users to make rigid assumptions, while there are still other advanced non-parametric methods should be considered. Secondly, DrGA deals with the censored data in a naive way, i.e., DrGA ignores missing survival information automatically, whereas, for end-of-study and loss-to-follow-up censoring, it selects the approach of analyzing dichotomized data. At last, we do not automate the data pre-processing procedure due to the heterogeneity of the data structure. However, the last restriction seems to be solved easily most as the users still may benefit partly from example codes provided in Additional file 1.

Moreover, because our desire is to see DrGA in the future become a focal point for the community of cancer researchers in analyzing driver genes comprehensively, we plan to continue to overhaul DrGA more. Specifically, we will first overcome the first limitation by integrating various correlation tests into DrGA and let DrGA choose an appropriate method for each certain case automatically. Also, although iWGCNA better performs its original version in terms of identifying biologically relevant functional modules, we understand that there has an absolute difference between clustering patients into different subgroups and clustering genes into different modules. Therefore, we have raised this point and proposed a novel tool named oCEM to overcome it, published elsewhere [35]. In the future, we will consider replacing iWGCNA with oCEM.

## Conclusions

In conclusion, we believe that the DrGA tool is a potential workaround for the non-technical users to efficiently implement complex analyses in R and gain reproducible and consistent results.

### Availability and requirements

- Project name: DrGA.
- Project home page: https://github.com/huynguyen250896/DrGA
- Operating system(s): Any.
- Programming language: R
- Other requirements: None.
- License: MIT.
- Any restrictions to use by non-academics: none.

## Supplementary Information

**Additional file 1:** User manual. Tutorial and use examples of DrGA

## Data Availability

The R package DrGA under the MIT license and R codes to reproduce all results shown in the study are made available freely on GitHub (https://github.com/huynguyen250896/DrGA). The users can download the raw data of human breast cancer from the cBioPortal for Cancer Genomics (http://www.cbioportal.org) [41, 42] under the accession number EGAS00000000083 and mouse metabolic syndrome data from the Gene Expression Omnibus (GEO; http://www.ncbi.nlm.nih.gov/geo) under the accession number GSE2814. Approval by a local ethics committee is not required, and all the data can be immediately downloaded for research purposes.

## Abbreviations

DrGA: DriverGeneAnalysis
oCEM: Overlapping CoExpressed gene Module
WGCNA: Weighted gene co-expression network analysis
iWGCNA: Improved weighted gene co-expression network analysis
HR: Hazard ratios
CI: Confidence intervals
