# Supplementary material for: DrGA: cancer driver gene analysis in a simpler manner
Source: BMC Bioinformatics. 2022 Mar 5;23:86. doi: 10.1186/s12859-022-04606-0 (PMC8897886; doi:10.1186/s12859-022-04606-0)
Supplement: Supplementary file 1 — Additional file 1: User manual. Tutorial and use examples of DrGA [file 12859_2022_4606_MOESM1_ESM.docx]

**Additional File 1**

**User Manual:**

**DrGA: cancer driver gene analysis in a simpler manner**

Quang-Huy Nguyen^1^ & Duc-Hau Le^1,2*^

^1^Department of Computational Biomedicine, Vingroup Big Data Institute, Hanoi, Vietnam.

^2^College of Engineering and Computer Science, VinUniversity, Hanoi, Vietnam.

* To whom correspondence should be addressed. Tel: (84)912324564; Email: [hauldhut@gmail.com](mailto:hauldhut@gmail.com)

Table of Contents

[I. Human breast cancer 3](#_Toc73712056)

[1. Identification of driver genes. 3](#_Toc73712057)

[2. Pre-processing procedures & Run DrGA 3](#_Toc73712058)

[3. Understanding the tool and gained results 6](#_Toc73712059)

[a. Results from the module 1 7](#_Toc73712060)

[b. Results from the module 2 7](#_Toc73712061)

[c. Results from the module 3 8](#_Toc73712062)

[d. Results from the module 4 11](#_Toc73712063)

[II. Mouse metabolic syndrome 16](#_Toc73712064)

[1. Description of the data 16](#_Toc73712065)

[2. Pre-processing procedures & Run DrGA 16](#_Toc73712066)

[Supplementary References 21](#_Toc73712067)

DrGA is an R package can be freely accessed on our Github (<https://github.com/huynguyen250896/DrGA>). The method has been developed based on the idea of our most recent driver gene analysis scheme (1). Before your raw data become inputs of DrGA, it requires two following steps: (i) Determine candidate driver genes using advanced driver gene identification tools, and (ii) Do pre-processing procedures to turn your raw data structure into a satisfactory structure (Figure S1).

To show a comprehensive picture of using DrGA, we re-use -omic data used in our prior study (1), downloaded from our github repository (<https://github.com/huynguyen250896/DrGA/tree/master/data_n_code/breast_cancer>) or the cBioPortal for Cancer Genomics (<http://www.cbioportal.org)> (2, 3), including somatic mutation (MUT; n = 2,369), gene expression (EXP; n = 1,904), and copy number alteration (CNA; n = 2,173), in a cohort of breast cancer patients. Due to as an application example, to simplify all the processes and be convenient to count the time as well as reproduce the results from the previous study (1), we decide to preserve the tools at each stage with their selected parameters as the previous study unless otherwise specified.

Next, we apply our tool to mouse metabolic syndrome containing liver EXP from female mice (n =135) (4). The raw data and R codes for pre-processing processes can be seen in (<https://github.com/huynguyen250896/DrGA/tree/master/data_n_code/metabolic_syndrome>).


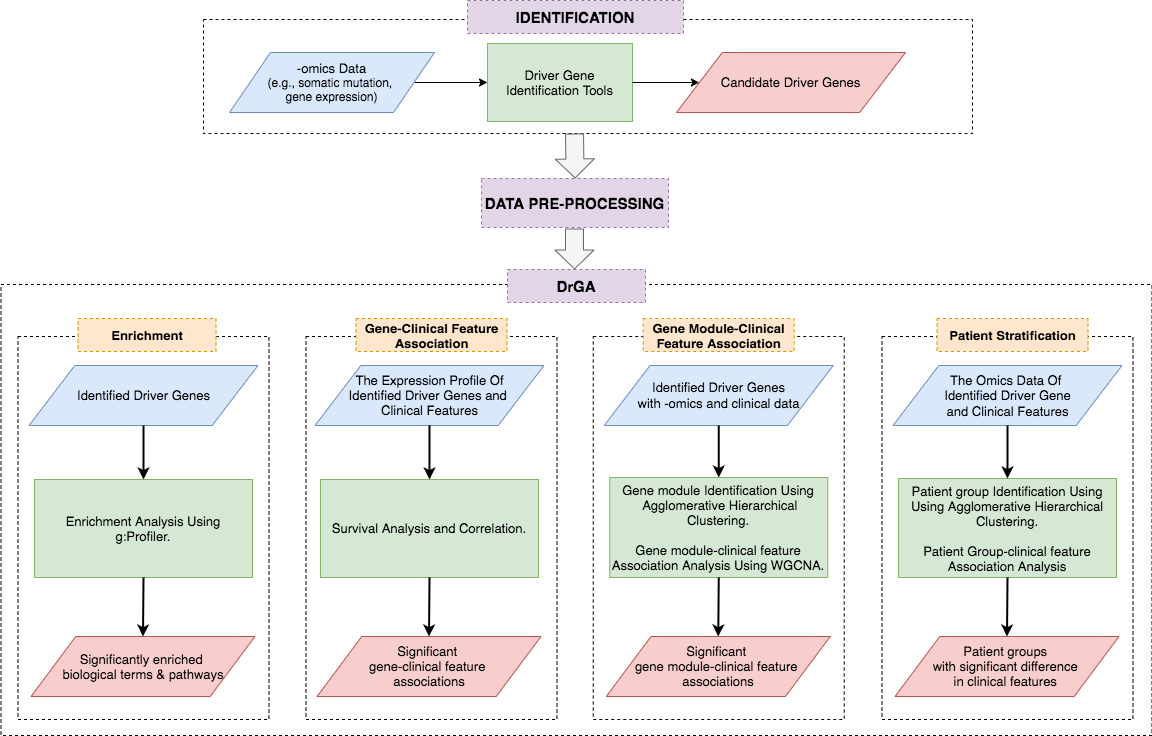


**Figure S1.** Users first use high-tech driver gene identification tools to predict cancer-related genes. Then, raw data must be processed following the data structure required by the tool DrGA before running the four analysis modules (left to right).

# I. Human breast cancer

## 1. Identification of driver genes.

In this step, we input MUT data into the two web-based tools OncodriveFML (5) and OncodriveCLUSTL (6), rendering a total of 35 unique driver genes, in which 30 and 10 driver genes were predicted by the two, respectively. We then match those 35 genes with three gene lists from the Cancer Gene Census database (<https://cancer.sanger.ac.uk/census>) (7), Pereira *et al*. reference paper (8) and Nik Zainal *et al.* paper (9), and realize that 31 out of 35 are genuine (Table S1).

| MAP2K4, ARID1A, PIK3CA, TBX3, MAP3K1, TP53, AKT1, GATA3, CDH1, RB1, CDKN1B, NCOR1, CDKN2A, ERBB2, KRAS, BRCA2, BAP1, PTEN, CBFB, KMT2C, RUNX1, NF1, PIK3R1, ERBB3, FOXO3, SMAD4, GPS2, AGTR2, ZFP36L1, MEN1, SF3B1. |
| --- |

**Table S1.** 31 validated driver genes are used for downstream analyses.

## 2. Pre-processing procedures & Run DrGA

Here the first module (i.e., enrichment analysis) only needs a list of the driver genes above (Table S1), so we will place it in the two input data (e.g., EXP and CNA) required for the three leftover modules. The second and third modules (i.e., association analyses) need the same input data (usually EXP selected). Finally, input data for the last module can be any data of your choice (e.g., EXP, CNA, methylation, ...). In particular, this work selects EXP for the second and third modules, and CNA for the fourth module as examples, the user can replace them with anything else if appropriate.

Firstly, we will set up the working directory, and call necessary R libraries. Set up the working directory is very important due to two reasons: (i) let you be able to import the raw data into R and (ii) the majority of results given by DrGA will move directly into. Secondly, we input the raw data (i.e., assigned as exp, cna, and clinical) into the R environment.

*#set up the working directory*

setwd("~/path/to/your/raw/data")

*#library*

devtools::install_github("huynguyen250896/DrGA", force = T) #NOTE: Download all dependencies of the tool!

x=c("DrGA", "dplyr", "survival", "tibble", "tidyr", "ComplexHeatmap",

'cluster', 'mclust', 'clValid', 'Biobase', 'annotate', 'GO.db',

'mygene', "dynamicTreeCut", "flashClust", "Hmisc", "WGCNA","purrr",

"gprofiler2", "table1", "compareGroups")

lapply(x, require, character.only = TRUE)

*#load raw data*

exp = read.table('data_mRNA_median_Zscores.txt', sep = '\t', check.names = FALSE, header = TRUE, row.names = NULL)

cna = read.table('data_CNA.txt', sep = '\t', check.names = FALSE, header = TRUE, row.names = 1)

clinical = read.table('data_clinical_patient.txt', sep = '\t', check.names = FALSE, header = TRUE, row.names = 1, fill=TRUE)

To meet the requirement of the first module, we simply put those 31 driver genes into exp and cna. As exp and cna have different dimensions, we will naturally have two corresponding clinical data for each (called clinicalEXP and clinicalCNA, respectively). Remember that exp and cna are two matrices whose rows are samples and columns are genes, whereas clinicalEXP and clinicalCNA include their rows are samples, and their columns are clinical features of the breast cancer patients.

*#identified driver genes*

driver=c("MAP2K4", "ARID1A", "PIK3CA", "TBX3", "MAP3K1", "TP53", "AKT1", "GATA3", "CDH1", "RB1", "CDKN1B", "NCOR1", "CDKN2A", "ERBB2", "KRAS", "BRCA2", "BAP1", "PTEN", "CBFB", "KMT2C", "RUNX1", "NF1", "PIK3R1", "ERBB3", "FOXO3", "SMAD4", "GPS2", "AGTR2", "ZFP36L1", "MEN1","SF3B1")

length(driver) #31 driver genes

*#only keep the 31 driver genes in exp and cna*

exp=exp %>%

dplyr::filter(.$Hugo_Symbol %in% driver) %>%

tibble::column_to_rownames('Hugo_Symbol') %>%

dplyr::select(-Entrez_Gene_Id)

cna=cna[driver, ]

*#check dimension*

dim(exp) # 31 1904

dim(cna) # 31 2173

dim(clinical) # 2509 21

*#match patients sharing between exp versus clinical, and cna versus clinical*

*#exp and cna are two matrices whose rows are samples and columns are genes*

exp = exp[,intersect(colnames(exp), rownames(clinical))] %>% t()

clinicalEXP = clinical[intersect(rownames(exp), rownames(clinical)), ]

cna = cna[,intersect(colnames(cna), rownames(clinical))] %>% t()

clinicalCNA = clinical[intersect(rownames(cna), rownames(clinical)), ]

To meet the requirement of the second and third modules, we use exp and clinicalEXP. For exp, its format is now ready to use. Note that, to prevent outliers in exp from negatively affecting co-expression network construction, we use hierarchical clustering method to identify and remove them. As a result, we do not detect any outliers and so do not show R code below. However, for the second example on mouse metabolic syndrome, the user can find out this process. For clinicalEXP, its columns must only possess clinical features of your choice. This study re-selects three clinical features like the number of lymph nodes lymph (continuous variable), Nottingham prognostic index npi (continuous variable), and tumor stage stage (ordinal variable). Since the second module performs correlation analyses between the expression levels of individual driver genes and each clinical feature, while the third module performs correlation analyses between the expression levels of individual functional modules and each clinical feature, we will assign variable type for the three clinical features in clinicalEXP as ‘numeric’ or ‘integer’ in R. In addition, to run survival analyses between the expression levels of individual driver genes and patient outcome, clinicalEXP must have two additional columns like overall survival time time (continuous variable) and overall survival status status (binary variable; usually coded 1 as death and 0 as alive) of all the subjects.

*#preprocess clinicalEXP*

clinicalEXP = clinicalEXP %>%

tibble::rownames_to_column('sample') %>%

dplyr::select(c(sample, LYMPH_NODES_EXAMINED_POSITIVE, NPI, stage, OS_MONTHS, OS_STATUS)) %>%

dplyr::mutate(status = ifelse(clinicalEXP$OS_STATUS == "DECEASED",1,0)) %>%

tibble::column_to_rownames('sample') %>%

dplyr::select(-OS_STATUS)

colnames(clinicalEXP)[1:4] = c("lymph", "npi", "stage", "time")

str(clinicalEXP)

*# 'data.frame': 1904 obs. of 5 variables:*

*# $ lymph : int 1 5 8 1 0 1 0 2 0 6 ...*

*# $ npi : num 4.04 6.03 6.03 5.04 3.05 ...*

*# $ stage : int 2 2 3 2 2 2 1 2 2 4 ...*

*# $ time : num 47 20.4 138.1 119.8 101.2 ...*

*# $ status: num 1 1 0 0 0 0 0 0 0 1 ...*

To meet the requirement of the last module, we do exactly the same as the second and third modules for cna and clinicalCNA. To help DrGA automatically detect which statistical test is appropriate for testing difference between identified patient subgroups in terms of each clinical feature in clinicalCNA, the user should carefully pre-define variable type of each in R. For example, lymph and npi are continuous variables should be assigned as a ‘numeric’ or ‘integer’ type in R, whereas stage is an ordinal variable should be assigned as a ‘character’ or ‘factor’ type in R.

*#preprocess clinicalCNA*

clinicalCNA = clinicalCNA %>%

tibble::rownames_to_column('sample') %>%

dplyr::select(c(sample, LYMPH_NODES_EXAMINED_POSITIVE, NPI, stage, OS_MONTHS, OS_STATUS)) %>%

dplyr::mutate(status = ifelse(clinicalCNA$OS_STATUS == "DECEASED",1,0)) %>%

tibble::column_to_rownames('sample') %>%

dplyr::select(-OS_STATUS)

colnames(clinicalCNA)[1:4] = c("lymph", "npi", "stage", "time")

clinicalCNA$stage = as.character(clinicalCNA$stage)

str(clinicalCNA)

*# 'data.frame': 2173 obs. of 5 variables:*

*# $ lymph : int 10 0 3 24 1 3 0 0 0 1 ...*

*# $ npi : num 6.04 2.04 5.04 6.07 4.05 ...*

*# $ stage : chr "2" "1" "2" "2" ...*

*# $ time : num 140.5 163.5 164.9 14.1 103.8 ...*

*# $ status: num 0 0 0 1 0 0 0 0 0 0 ...*

Now, put all the processed data into the function DriverGeneAnalysis in the R package DrGA and run.

*#RUN!!!!*

*#make sure that patients that share between exp and clinicalEXP are #included at their rows and in exactly the same order*

all(rownames(exp) == rownames(clinicalEXP))

*#[1] TRUE*

*#make sure that patients that share between cna and clinicalCNA are #included at their rows and in exactly the same order*

all(rownames(cna) == rownames(clinicalCNA))

*#[1] TRUE*

drga = DriverGeneAnalysis(exp = exp, clinicalEXP = clinicalEXP, timeEXP = clinicalEXP$time, statusEXP = clinicalEXP$status,

datMODULE4 = cna, cliMODULE4 = clinicalCNA, timeMODULE4 = clinicalCNA$time, statusMODULE4 = clinicalCNA$status)

where the argument exp is the place where the processed data exp are inputted to serve to run the second and third modules of DrGA. Its corresponding clinical data clinicalEXP, overall survival time clinicalEXP$time, and overall survival status clinicalEXP$status of all the patients are inputted into the three arguments clinicalEXP, timeEXP, and statusEXP, respectively, to serve to perform correlation analyses in these modules. Similarly, the argument datMODULE4 is the place where the processed data cna are inputted to serve to run the last module. Its corresponding clinical data clinicalCNA, overall survival time clinicalCNA$time, and overall survival status clinicalCNA$status of all the patients are inputted into the three arguments cliMODULE4, timeMODULE4, and statusMODULE4, respectively, to serve to perform survival analysis as well as observe statistically significant differences between identified patient subgroups in terms of clinical features of your interest.

If the tool runs smoothly and successfully, several results will be printed out in the R environment, and some will be moved directly to the working directory. We show them in the section ‘Understanding the tool and gained results’ below. The user can find the results of this example running at <https://github.com/huynguyen250896/DrGA/blob/master/data_n_code/breast_cancer/output_BRCA.zip>.

## 3. Understanding the tool and gained results

Since DrGA includes state-of-the-art statistical tools; specifically, g:Profiler (10) for the first module, computeC (1) and geneSA (1) for the second module, WGCNA (11) with several improvements (1) for the third module, and hierarchical agglomerative clustering (12) for the last module, we suggest the user to refer to their original papers to grasp how to interpret results well. Alternatively, the user can also refer to our study (1). Here we outline the interpretation of them.

### a. Results from the module 1

The results of the first module will move to the working directory as a txt file named *EnrichmentAnalysis.txt*. This contains the column ‘source’ reports types of selected biological mechanisms: biological processes (GO:BP) and KEGG pathways (KEGG), while the column ‘term_name’ points out certain biological mechanisms. g:Profiler then tests the statistical significance among all results and shows at the column ‘p_value’ (g:SCS multiple testing correction method (10); the smaller the P-value, the more significant). Finally, we can know which gene specifically involves which biological mechanisms and how many through the ‘intersections’ and ‘query_size’ columns, respectively. Note that, DrGA may annotate driver genes from other species rather than humans by using the argument organism. Please refer to a full list of organisms at (<https://biit.cs.ut.ee/gprofiler/page/organism-list>). In addition, the argument sources helps you choose possible biological mechanisms of driver genes (e.g., Gene Ontology - 'GO:BP', 'GO:MF', 'GO:CC'; 'KEGG'; 'REAC'; 'TF'; 'MIRNA'; 'CORUM'; 'HP'; 'HPA'; 'WP';…).

### b. Results from the module 2

The result from successfully performing the association analysis between the expression of individual driver genes and survival rates of all the patients is reported in *gene_SA.txt* in the working directory (Table S2). Roughly, given a driver gene, the median expression of that gene was calculated across the patients, then the patients were classified into two groups based on the expression of the gene. The first group ‘up-regulation’ includes patients having the expression of the genes was greater than the median; meanwhile, the second group ‘down-regulation’ includes patients having the expression of the genes was less than the median. The column ‘HR’ implies the Hazard ratio with its 95% confidence interval (95% CI) that is a measure that helps to determine whether either of two expression levels of each driver gene will result in an increased (i.e., HR > 1) or decreased (i.e., HR < 1) probability of experiencing the defined event (i.e., death), at any time (in this case, the below-median expression level is the reference). P-values are computed by the Cox proportional hazard method to test the statistical difference between the given results. Q-value is computed following the Benjamini–Hochberg procedure (13).

| **Gene** | **HR (95% CI)** | **P-value** | **Q-value** |
| --- | --- | --- | --- |
| AKT1 | 1.30 (1.15-1.46) | 1.48×10^-05^ | 2.29×10^-04^ |
| KMT2C | 1.24 (1.10-1.40) | 3.47×10^-04^ | 2.69×10^-03^ |
| KRAS | 1.20 (1.07-1.35) | 2.30×10^-03^ | 1.19×10^-02^ |
| PTEN | 0.85 (0.76-0.96) | 7.92×10^-03^ | 2.73×10^-02^ |
| TBX3 | 0.84 (0.75-0.95) | 4.91×10^-03^ | 1.90×10^-02^ |
| PIK3R1 | 0.84 (0.75-0.95) | 4.37×10^-03^ | 1.93×10^-02^ |
| MAP3K1 | 0.82 (0.73-0.93) | 1.23×10^-03^ | 7.61×10^-03^ |
| SMAD4 | 0.78 (0.69-0.88) | 4.85×10^-05^ | 5.01×10^-04^ |
| MAP2K4 | 0.76 (0.67-0.85) | 4.57×10^-06^ | 1.42×10^-05^ |

**Table S2.** **Association between the expression of individual driver genes and the overall survival of BRCA patients**. Three genes including *AKT1, KMT2C,* and *KRAS* with above-median expression level and six genes including *PIK3R1, PTEN, SMAD4, MAP3K1, MAP2K4* and *TBX3* with below-median expression level significantly associated with a shortened lifespan. HR: hazard ratio. 95% CI: 95% confidence interval.

The result from successfully performing the association analysis between the expression of individual driver genes and each clinical feature is reported as a txt file termed *CC_results.txt* in the working directory. The Spearman’s rank-order correlation analysis (default in DrGA) is computed by DrGA between the expression of individual driver genes and each leftover clinical feature (i.e., numbers of lymph nodes, Nottingham prognostic index, and pathologic stage). The user can use other correlation methods with the argument methodCC (e.g., Pearson’s correlation - ‘pearson’ or Kendall’s correlation - ‘kendall’). Table S3 reports correlation coefficients *r* (column ‘CC’), P-values, and Q-values of each driver gene with all the three clinical features. The column ‘CC’ measures the degree of association between the two variables: the expression levels of each driver gene versus each clinical feature. It takes on values ranging between -1 and +1. When *r* = 0, there is no relationship between the two variables. When *r* closer to 1, there is an increasingly strong positive (uphill) relationship between the two variables, otherwise is an increasingly strong negative (downhill) relationship between the two variables. Q-value is computed following the Benjamini–Hochberg procedure.

| **Gene** | **Number of lymph nodes** | | | **Nottingham prognostic index** | | | **Cancer Stage** | | |
| --- | --- | --- | --- | --- | --- | --- | --- | --- | --- |
|  | **CC** | **P-value** | **Q-value** | **CC** | **P-value** | **Q-value** | **CC** | **P-value** | **Q-value** |
| ARID1A | -0.06 | 0.01 | 0.02 | -0.13 | 1.31×10^-8^ | 3.20×10^-8^ | -0.10 | 1.13×10^-4^ | 4.73×10^-4^ |
| RUNX1 | -0.14 | 1.65×10^-9^ | 3.63×10^-8^ | -0.25 | 2.20×10^-16^ | 2.42×10^-15^ | -0.11 | 2.97×10^-5^ | 3.12×10^-4^ |
| GATA3 | -0.01 | 1.27×10^-5^ | 9.31×10^-5^ | -0.28 | 2.20×10^-16^ | 4.84×10^-15^ | -0.12 | 3.83×10^-5^ | 2.68×10^-4^ |
| TBX3 | -0.10 | 9.10×10^-6^ | 1.00×10^-4^ | -0.18 | 1.44×10^-15^ | 6.31×10^-15^ | -0.12 | 6.12×10^-5^ | 3.21×10^-4^ |
| NF1 | -0.09 | 5.77×10^-5^ | 3.17×10^-4^ | -0.08 | 2.23×10^-4^ | 3.07×10^-4^ | -0.07 | 6.36×10^-3^ | 0.02 |
| MAP2K4 | -0.08 | 4.61×10^-4^ | 1.69×10^-3^ | -0.22 | 2.20×10^-16^ | 1.21×10^-15^ | -0.07 | 6.99×10^-3^ | 0.02 |
| PTEN | -0.08 | 6.02×10^-4^ | 1.89×10^-3^ | -0.23 | 2.20×10^-16^ | 1.61×10^-15^ | -0.11 | 2.93×10^-5^ | 6.15×10^-4^ |
| SMAD4 | -0.06 | 0.01 | 0.03 | -0.10 | 1.79×10^-5^ | 3.29×10^-5^ | -0.08 | 1.48×10^-3^ | 3.89×10^-3^ |
| MAP3K1 | -0.06 | 0.01 | 0.03 | -0.16 | 6.35×10^-13^ | 2.00×10^-12^ | -0.09 | 7.16×10^-4^ | 2.15×10^-3^ |
| SF3B1 | 0.09 | 7.83×10^-5^ | 1.02×10^-3^ | 0.07 | 1.78×10^-3^ | 3.31×10^-3^ | 0.10 | 8.48×10^-5^ | 1.19×10^-3^ |

**Table S3. Association between the expression of driver genes and the other clinical features.** *ARID1A, RUNX1, GATA3, TBX3, NF1, MAP2K4, PTEN, SMAD4, MAP3K1* and *SF3B1* significantly associated with all of the three clinical features. CC: correlation coefficient.

Alternatively, the user also can see specifically the results in the txt file *CC_results.txt* in R by command

View(drga$CC_module2)

### c. Results from the module 3

When DrGA goes to the third module, it will ask the user to choose a value of soft-thresholding power based on the Figure S2 (shown in the R environment). The idea behind soft thresholding is to emphasize more on stronger associations (larger *r*). Based on a recommendation from the previous study (1), you should choose a point at which R^2^ reaches the peak for the first time (y-axis). Namely, we select six for this case.

**Figure S2.** The graph printed out in the R environment shows the scale-free fit index (y-axis) as a function of the soft-thresholding power β (x-axis).

In the middle of the analysis, we will see that DrGA automatically recognizes the best agglomeration method for grouping driver genes into functional modules (Figure S3; printed out in the R environment). More specifically, a set of the agglomeration methods, including ‘complete’, ‘average’, ‘single’, or ‘ward’, will be reviewed and picked the best one by DrGA.


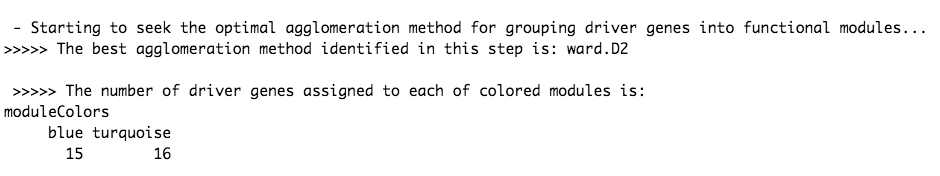


**Figure S3.** DrGA automatically detects the ward’s hierarchical clustering is the best method, and then indicates two functional gene modules (marked as blue and turquoise) along with the number of driver genes distributed into each.

Note that, as a recommendation from the previous study (1), we recommend choosing the ten number of genes existing in each module minimally (default in DrGA) but the user can set a different number with the argument minClusterSize (e.g., minClusterSize = 100). Besides, DrGA constructs a ‘signed’ network as default but it also can be ‘unsigned’ or ‘signed hybrid’ network, modified by the argument NetworkType (e.g., NetworkType = 'unsigned '). In addition, the user also may want to see specifically which genes are assigned to which module by command:

View(drga$moduleColors_module3)

Figure S4 (*Dendro-MolduColor.pdf* in the working directory) illustrates the co-expressed modules highlighted with distinct colors, and the dendrogram height computed by one minus Pearson’s correlation values (1-*r*) represents dissimilarity of two driver genes, in which low dissimilarities indicate that two driver genes are close (similar), whereas the high dissimilarities imply two driver genes are far apart (dissimilar). When it comes to the color of modules, the grey color reserved for genes that do not belong to any identified modules and are not co-expressed as well, whereas the others dedicated to genes that do have their own functional module and are co-expressed.

**Figure S4.** Dendrogram of the identified driver genes on Topology Overlap Matrix-based dissimilarity**.** The dendrogram height corresponds to the coefficient of dissimilarity matrix that is for every pair of the 31 driver genes, in which the low dissimilarities indicate two driver genes are close, whereas the high dissimilarities imply two driver genes are distant apart. Two co-expressed modules were detected and are shown in different colors.

Figure S5 (*Assoc-CliModul.pdf* in the working directory) shows the module-trait heatmap that represents the correlation of the modules with clinical features. The correlation coefficients *r* are computed following Pearson’s correlation with an interpretation that can be found in the above sub-section ‘Results from the module 2’.

**Figure S5.** Module–feature associations. Each row corresponds to a module eigengene (ME), column to a feature. Each cell contains the corresponding correlation coefficient *r* and P-value. lymph: number of positive lymph nodes, and npi: the Nottingham prognostic index.

DrGA automatically detects the top five intramodular hub genes in each co-expressed module (Figure S6; printed out in the R environment), indicating possession of a vast range of interactions with other genes as well as playing a crucial role in the co-expression network of those genes.


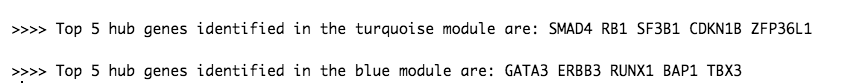


**Figure S6.** Top five hub genes in each identified module are printed out in the R environment.

### d. Results from the module 4

Here, DrGA automatically re-seeks the best agglomeration method for grouping breast cancer patients into distinct subgroups (Figure S7; printed out in the R environment).


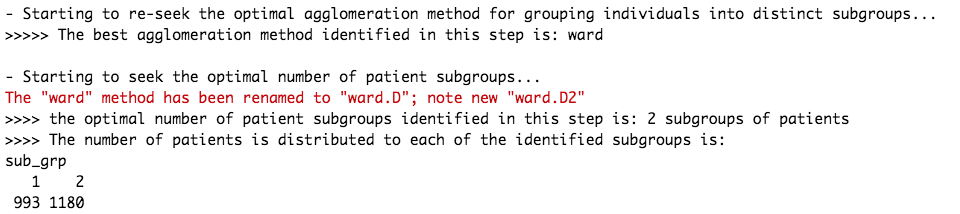


**Figure S7.** DrGA automatically indicates the ward’s hierarchical clustering is the best method once again, and then indicates two patient subgroups are the best along with the number of breast cancer patients distributed into each.

Here the user may see specifically which samples are assigned to which subgroup by R command:

View(drga$subgroups_module4)

To automatically determine the optimal number of patient subgroups, DrGA performs three common indices like the connectivity (Figure S8), the Dunn’s index (Figure S9) and the average Silhouette width (Figure S10), rendering *optimal-group-number.pdf* in the working directory.

**Figure S8.**  Two optimal groups were determined by the connectivity. The connectivity computes the degree of connectedness of a given group partitioning. The connectivity shows the connectedness of a given cluster partitioning and has a value between 0 and infinity. The user should choose a point reaching the most minimized value (y-axis).

**Figure S9.**  Two optimal groups were also determined by the Dunn’s index. The Dunn’s index (y-axis) has a value between zero (poorly clustered observations) and infinity (well clustered observations), and the place where the black line of Dunn’s index plot peaks at, which implies that that group number is optimal.

**Figure S10.** Three optimal groups were determined by the Silhouette width. The average Silhouette has a value between -1 (poorly clustered observations) and 1 (well clustered observations), and the place where the black line of the Silhouette plot peaks at, which implies that that group number is optimal.

The heatmap (Figure S11; *heatmap.pdf* in the working directory) shows the difference in the CNV events between the identified patient groups. If due to the large number of driver genes leading to impossibly showing gene names in rows of the heatmap, the user turns them off by using the logical argument hm_row_names (hm_row_names = F).

**Figure S11.** ward’s hierarchical clustering of breast cancer patients based on the 31 driver genes. Two distinct groups are found (marked as black and green). For CNA scale, Dark red, red, grey, blue and dark blue represent high-level amplification, amplification, copy‐neutral, deletion and high-level deletion, respectively.

DrGA next automatically implements survival analysis between the identified subgroups, and prints Cox P-value and the HR with its 95% CI out in the R environment (Figure S12). Interpretation of the HR can be found above. As shown in Figure S12, we conclude that the tumors in the second group exhibit significantly worse outcomes (HR is 1.294 with 95% CI (1.151–1.454), P-value < 0.01; group 1 is the reference).


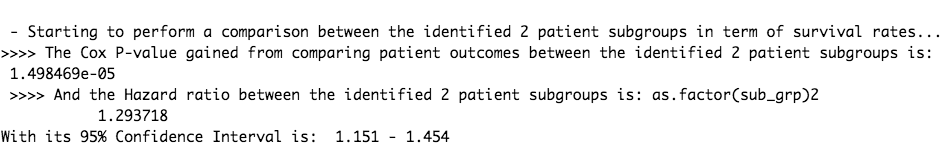


**Figure S12.** Cox P-value and HR with 95% CI are shown in R.

At last, DrGA automatically performs comparisons between the identified subgroups in terms of the three remaining clinical features (i.e., numbers of lymph nodes, Nottingham prognostic index, and pathologic stage) using statistical tests. The results are moved into the working directory as a xlsx file termed *tableSTAT.xlsx* (Table S4). Noticeably, depending on whether these clinical features are automatically defined by DrGA as continuous normal-distributed (i), continuous non-normal distributed (ii) or categorical (iii), the following descriptives and tests are performed.

- (i) is applied t-test or ANOVA
- (ii) is applied Kruskall-Wallis test
- (iii) is applied Chi-square or Fisher’s exact test

For instance, DrGA tells us about this information in the R environment, as illustrated in Figure S13.


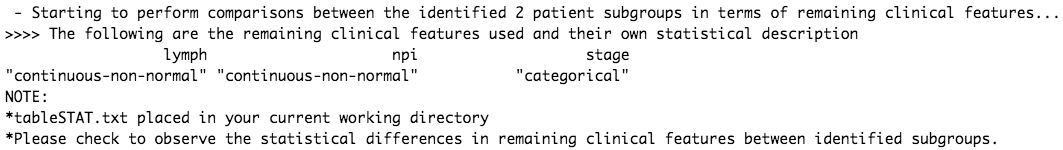


**Figure S13.** The statistical description of the clinical features. In this case, we understand that DrGA applies Kruskall-Wallis test to the first two variables lymph and npi, whereas Chi-square test is applied to stage.

|  | **1 (N=954)** | **2 (N=1002)** | **p-value** |
| --- | --- | --- | --- |
| lymph | 0.00 [0.00;2.00] | 0.00 [0.00;2.00] | 0.031 |
| npi | 4.03 [3.03;4.11] | 4.05 [3.08;5.05] | <0.001 |
| stage: |  |  | 0.016 |
| 0 | 8 (1.11%) | 8 (0.96%) |  |
| 1 | 270 (37.4%) | 258 (31.1%) |  |
| 2 | 393 (54.4%) | 479 (57.7%) |  |
| 3 | 45 (6.23%) | 81 (9.76%) |  |
| 4 | 6 (0.83%) | 4 (0.48%) |  |

**Table S4.** Comparison between the involved subgroups in terms of the chosen clinical features. For the first two continuous variables lymph and npi, median [percentiles 25%; percentiles 75%] are calculated at the first and second columns. For the ordinal variable stage, the number of cases and the percentage of cases in each tumor stage are shown. lymph: numbers of lymph nodes, npi: Nottingham prognostic index, stage: tumor stages.

# II. Mouse metabolic syndrome

## 1. Description of the data

The data is about female mice of a specific F2 intercross with metabolic syndrome (obesity, insulin resistance, dyslipidemia) that EXP measurements from livers. In the original paper (4), the authors have filtered from over physiologically related 20,000 genes by retaining only the most variant and most connected probes, rendering 3600 ones.

## 2. Pre-processing procedures & Run DrGA

Firstly, we load the raw data including EXP and its clinical data (exp and cli)

*#load raw file*

exp = read.table("LiverFemale3600.csv", header = T, check.names = F, sep=",")

cli = read.table("ClinicalTraits.csv", header = T, check.names = F, sep=",", row.names = 1)

We remove missing genes (i.e., coded as 0) and duplicated genes due to insufficient information to retain them.

*#remove missing gene names and duplicated genes in exp*

exp = exp[which(exp$gene_symbol != "0"),]

dup = duplicated(exp$gene_symbol)

exp = exp[which(dup == FALSE),]

*#turn exp1 into satisfactory format of DrGA*

*#DrGA requires data whose rows are samples and columns are genes.*

exp = exp %>%

dplyr::select(-c(substanceBXH, LocusLinkID, ProteomeID, cytogeneticLoc,

CHROMOSOME, StartPosition, EndPosition)) %>%

tibble::remove_rownames() %>%

tibble::column_to_rownames('gene_symbol') %>%

drop_na() %>% t()

Since the process of the gene co-expression network construction is very sensitive to outliers, we find potential ones by using the hierarchical clustering method. Finally, we exclude a mouse named F2_221 (Figure S14).

*#detect outliers*

sampleTree = hclust(dist(exp), method = "average")

par(cex = 0.6);par(mar = c(0,4,2,0))

plot(sampleTree, main = "Sample clustering to detect outliers", sub="", xlab="",

cex.lab = 1.5,cex.axis = 1.5, cex.main = 2)

*# Plot a line to show the cut*

abline(h = 12.2, col = "red");

*# Determine cluster under the line*

clust = cutreeStatic(sampleTree, cutHeight = 12.2, minSize = 10)

table(clust)

*# clust 1 contains the samples we want to keep.*

keepSamples = (clust==1)

exp = exp[keepSamples, ]

**Figure S14.** Detect and remove outliers.

We keep mice that share between exp and cli at their rows and in exactly the same order. Due to a large number of clinical features in the cli data, we also only keep eight nescessary clinical features among 20 physiological features; i.e., body weight weight_g, body length length_cm, abdominal fat ab_fat, total fat total_fat, ulcerative colitis UC, free fatty acids FFA, glycemic index Glucose, two LDL and VLDL cholesterol levels LDL_plus_VLDL (Figure S15).

*#match mouses that share between cli versus exp*

cli = cli[cli$Mice %in% rownames(exp),]

cli = cli %>%

remove_rownames() %>%

tibble::column_to_rownames('Mice') %>%

dplyr::select(-c(Number, sex, Mouse_ID, Strain, DOB, parents, Western_Diet,

Sac_Date, comments, Note))

*#how the clinical traitsrelate to the sample dendrogram.*

*# Re-cluster samples*

sampleTree2 = hclust(dist(exp), method = "average")

*# Convert traits to a color representation: white means low, red means high, grey means missing entry*

traitColors = numbers2colors(cli, signed = FALSE);

*# Plot the sample dendrogram and the colors underneath.*

plotDendroAndColors(sampleTree2, traitColors,groupLabels = names(cli),

main = "Sample dendrogram and trait heatmap")

*#white means a low value, red a high value, and grey a missing entry.*

**Figure S15.** How the clinical features relate to the sample dendrogram. White means a low value, red a high value, and grey a missing entry

*#Only keep several clinical features with red color*

cli = cli %>%

dplyr::select(weight_g, length_cm, ab_fat,

total_fat, UC, FFA, Glucose,

LDL_plus_VLDL)

*#make sure that mice that share between exp and cli are included at their rows and in exactly the same order*

all(rownames(exp) == rownames(cli))

*#[1] FALSE*

exp = exp[rownames(cli), ]

*#check dimension*

dim(exp)

*#[1] 134 2281*

dim(cli)

*#[1] 134 8*

#RUN!!!!

drga = DriverGeneAnalysis(exp = exp, clinicalEXP = cli, datMODULE4 = exp, cliMODULE4 = cli, organism = 'mmusculus', hm_row_names = F)

# Supplementary References

1. Nguyen Q-H, Le D-H. Improving existing analysis pipeline to identify and analyze cancer driver genes using multi-omics data. Scientific Reports. 2020;10(1):20521.

2. Cerami E, Gao J, Dogrusoz U, Gross BE, Sumer SO, Aksoy BA, et al. The cBio Cancer Genomics Portal: An Open Platform for Exploring Multidimensional Cancer Genomics Data. Cancer Discovery. 2012;2(5):401-4.

3. Gao J, Aksoy BA, Dogrusoz U, Dresdner G, Gross B, Sumer SO, et al. Integrative analysis of complex cancer genomics and clinical profiles using the cBioPortal. Science signaling. 2013;6(269):pl1.

4. Ghazalpour A, Doss S, Zhang B, Wang S, Plaisier C, Castellanos R, et al. Integrating Genetic and Network Analysis to Characterize Genes Related to Mouse Weight. PLOS Genetics. 2006;2(8):e130.

5. Mularoni L, Sabarinathan R, Deu-Pons J, Gonzalez-Perez A, López-Bigas N. OncodriveFML: a general framework to identify coding and non-coding regions with cancer driver mutations. Genome Biology. 2016;17(1):128.

6. Arnedo-Pac C, Mularoni L, Muiños F, Gonzalez-Perez A, Lopez-Bigas N. OncodriveCLUSTL: a sequence-based clustering method to identify cancer drivers. Bioinformatics. 2019;35(22):4788-90.

7. Futreal PA, Coin L, Marshall M, Down T, Hubbard T, Wooster R, et al. A census of human cancer genes. Nat Rev Cancer. 2004;4(3):177-83.

8. Pereira B, Chin S-F, Rueda OM, Vollan H-KM, Provenzano E, Bardwell HA, et al. The somatic mutation profiles of 2,433 breast cancers refine their genomic and transcriptomic landscapes. Nature Communications. 2016;7(1):11479.

9. Nik-Zainal S, Davies H, Staaf J, Ramakrishna M, Glodzik D, Zou X, et al. Landscape of somatic mutations in 560 breast cancer whole-genome sequences. Nature. 2016;534(7605):47-54.

10. Raudvere U, Kolberg L, Kuzmin I, Arak T, Adler P, Peterson H, et al. g:Profiler: a web server for functional enrichment analysis and conversions of gene lists (2019 update). Nucleic Acids Res. 2019;47(W1):W191-W8.

11. Langfelder P, Horvath S. WGCNA: an R package for weighted correlation network analysis. BMC Bioinformatics. 2008;9(1):559.

12. Lance GN, Williams WT. A General Theory of Classificatory Sorting Strategies: 1. Hierarchical Systems. The Computer Journal. 1967;9(4):373-80.

13. Benjamini Y, Hochberg Y. Controlling the False Discovery Rate: A Practical and Powerful Approach to Multiple Testing. Journal of the Royal Statistical Society Series B (Methodological). 1995;57(1):289-300.
